# Analysis of household LPG demand elasticity in Cameroon and policy implications

**DOI:** 10.1016/j.heliyon.2023.e16471

**Published:** 2023-05-25

**Authors:** Flavian Emmanuel Sapnken, Marius Tony Kibong, Jean Gaston Tamba

**Affiliations:** a Laboratory of Technologies and Applied Science, IUT Douala, University of Douala, PO Box 8698, Douala, Cameroon; b Transports and Applied Logistics Laboratory, University Institute of Technology, University of Douala, PO Box 8698, Douala, Cameroon; c Energy Insight-Tomorrow Today, PO Box 2043, Douala, Cameroon

**Keywords:** LPG consumption, Price and income elasticities, Urbanization, Cameroon

## Abstract

Liquefied petroleum gas (LPG) is rapidly becoming a key part of Cameroon's energy mix, with enormous future potential. However, there are still many uncertainties about the extent of its potential market, which has so far often led to supply shortages. These shortages therefore constitute a major obstacle to the objective of promoting LPG as the main fuel in Cameroonian households. Accordingly, the short and long-run elasticities of LPG consumption in Cameroon are investigated in this work. This work uses annual time series data from 1994 to 2017. A basic model and four alternative specifications are used. Mid-run price and income elasticities of LPG consumption are found to be between −0.330 and −0.401, and, 0.159 and 0.569, respectively. Of all the five models, the error correction model is the most robust and the elasticities estimates reveal that price, income and urbanization are important determinants of LPG consumption in Cameroon. These results are consistent with those given by the other models, and in line with previous research on developing countries whose economic and demographic situation is similar to that of Cameroon. These results have serious implications on demand side management and calls for policy makers to promote widespread use of LPG especially in the savannah zone in order to reduce deforestation and overdependence on biomass.

## 1 Introduction

Cameroon began a reform of its oil products (OP) tax on January 1, 2017, which resulted in an increase in the consumption taxes on diesel from 0.15 USD/liter to 0.24 USD/liter and from 0.086 USD/liter to 0.12 USD/liter on gasoline consumption^1^ [1]. Although these tax increase is seen as a significant development after a decade of debate regarding the reform of the OP tax in Cameroon, this reform is modest as the majority of the OP tax is primarily used to fund road maintenance through the Road Usage Renewal system, which is based on the idea that users of a network should pay for its upkeep [2]. The Cameroonian government's determination to regulate OP consumption, where rising demand has given way to worries about global warming, local pollution, and oil security, among others, is at least revealed by the OP tax reform, despite its relatively small scale.

Since Cameroon joined the closed circle of liquefied natural gas producers in 2018 [1], some of the gas is processed locally and served to households via marketers.^2^ However, this is not enough to satisfy local demand for LPG. As a result, the country is forced to resort to imports. To cover its needs in 2021, Cameroon planned to import 120,000 metric tonnes (MT) of LPG. Despite this, households have been facing LPG scarcity in several localities in Cameroon (especially in urban areas) for a couple of years now. In September 2022, the government confessed that this shortage was due to the growing weight of the state subsidy dedicated to maintaining LPG prices. LPG subsidy is supported by the Hydrocarbon Price Stabilization Fund (HPSF).

At the end of March 2022, the Ministry of Commerce estimated the subsidy for a cylinder of gas at 10.17 USD and the HPSF projected the annual cost of this subsidy at 105 millions USD. This was more than its overall costs, which were estimated at 99 millions USD in 2021 and 78 millions USD in 2020. However, the Cameroon Company of Petroleum Deposits (CCPD) predicted that by 22 August 2022, the country will have two weeks of LPG autonomy.^3^

### 1.1 Preceding studies and identification of gaps

In the literature, there are many different methods for estimating the demand for energy, ranging from simultaneous model structures to aggregate analyses of the link between demand, income, and pricing [[3], [4], [5], [6]], to more detailed disaggregated analysis [[7], [8], [9]].

There aren't many studies on energy consumption in underdeveloped economies, and those that do tend to ignore the time-series aspects of how energy use adjusts with prices and wealth. Additionally, the Middle East [10,11], Latin America [[12], [13], [14]], and Asia [[15], [16], [17], [18]] have received the majority of attention in studies on energy demand functions. We do observe that there is a paucity of research on household fuels in low- and middle-income nations. Studies like those by Athukorala and Wilson [19] in Sri Lanka, Atakhanova and Howie [20] in Kazakhstan, and Filippini and Pachauri [21] in India have focused on the demand for electricity alone.

Although more accurate and robust assesment of energy consumption drivers are essential to improve implementation of energy policies and decision-making, studies in sub-Saharan Africa, especially Cameroon, are scarce. Furthermore, only a small portion of the sparse literature incorporates structured econometric analyses of how fluctuations in income, price and other reference factors affect energy consumption. To the best of authors’ knowledge, Nkutchet [22] is the only source that even vaguely reports estimates of the national LPG elasticity for the period 1975–1994, mostly from a statistical rather than an economic perspective. Energy demand is often modelled using economic data to reveal income and price elasticity. However, this cannot be accurate unless the scale and structure of the data collection samples are taken into account, as well as the nature of the time series in the study.

Many related studies (such as Gately and Huntington [5]) have used prices reported in the world market instead of domestic prices. This approach can, however, produce spurious results, as subsidy levels, cross-subsidy programmes, consumption taxes and local import taxes may prevent most consumers from being exposed to changes or variations in world market prices [23]. Using mean prices instead of spot prices is another major shortcoming in many energy studies. In order to accurately reflect the charasteristics of the link relating income and price of LPG demand, we use actual LPG prices on spot markets.

The key role of LPG demand in the Cameroonian economy, along with concerns about setting appropriate tariffs and the dual issue of increasing local consumption in national oil production, added to the country's energy deficits, all point to a clear need for robust elasticities estimates. However, access to high-quality data is a serious obstacle to LPG sector analysis in Cameroon. By conducting the first (as far as we know) econometric analysis of the demand function for LPG in Cameroon's households using recent data covering a relatively longer period (1994–2017), we begin to fill this gap in the research. In several respects therefore, this contribution builds on what has been done previously.

### 1.2 Objective, contribution and novelty

It is crucial to understand the relationships between LPG demand, price, disposable income and order socio-economic indicators in order to evaluate how well Cameroon's tax reform has altered LPG consumption. Additionally, this insight has major repercussions for energy policy.

Accordingly, this study's main goal is to estimate the dynamics of LPG demand in Cameroon using econometric models to derive robust elasticity estimates. In doing so, we seek to determine whether LPG demand and its drivers are linked in the long run. This empirical study aims to close a significant gap in previous research on less developed economies. This paper is the first comprehensive econometric analysis of the LPG demand function in Cameroon. In addition, this study examines the properties of time series so as to minimize erroneous outcomes arising from a non robust method. The study's data spans the period 1994–2017.

## 2 Energy demand trends in Cameroon's households

**Fig. 1:**
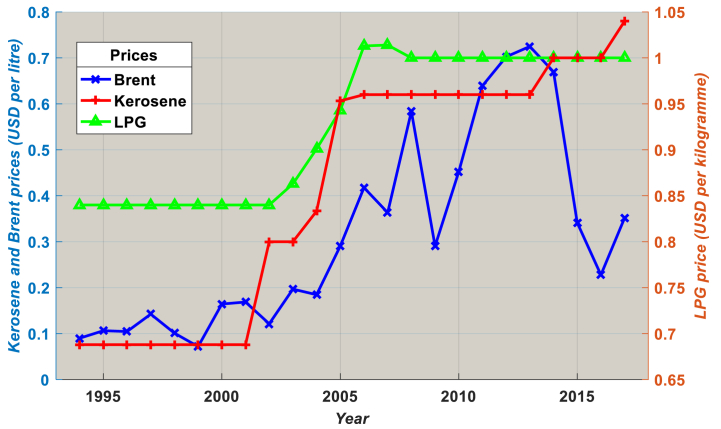
Real Brent, LPG and kerosene prices from 1994 to 2017. Source: OPEC [26]. Notes: The national average LPG and kerosene prices are the same in all 10 regions of Cameroon. This is because HPSF ensures that prices are equalized and harmonized across the country.

Fig. 1 displays the Brent, LPG, and kerosene prices from 1994 to 2017. With the exception of a few short periods, domestic kerosene and LPG prices mostly follow movements in the price of Brent. Although the government controls Cameroon's domestic LPG prices, they are frequently revised based on the cost of Brent on the world market. As seen in Fig. 1, the prices of LPG and kerosene frequently follow that of Brent on a global scale. The price of LPG was initially liberalized in 2002, followed by liberalizations in 2007 and 2008, among other key improvements [24]. Based on the weighted average price change of various international exchanges, the government, via the Hydrocarbon Price Stabilization Fund (HPSF), frequently establishes a reference price for crude oil on an ad hoc basis. The decision-making power for wholesale and retail LPG ex-plant rates is subsequently entrusted to HPSF, although the fuel for local oil firms, who manage the retail marketers, comes from CCPD [25]. Values for the marketers are not allowed to vary from those fixed by HPSF. This is why the prices of kerosene and LPG often remain unchanged throughout the whole country (and sometimes for several years).

**Fig. 2:**
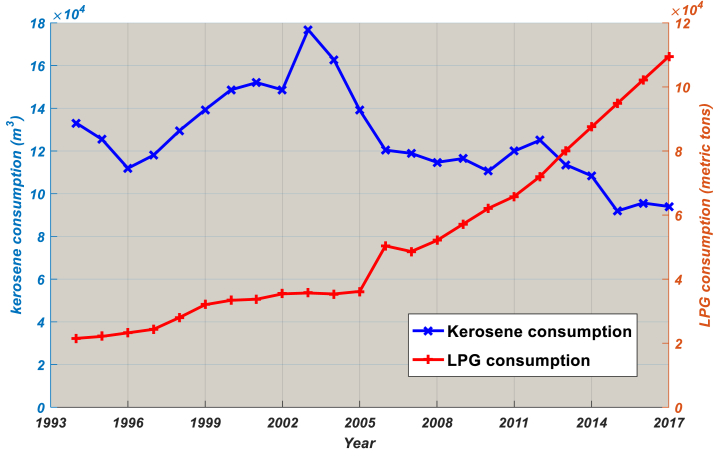
Historical LPG and kerosene consumption, 1994–2017. Source: Ministry of Energy [27]. Notes: The consumption of LPG and kerosene in all 10 regions is calculated by adding up each region's individual consumption.

**Fig. 3:**
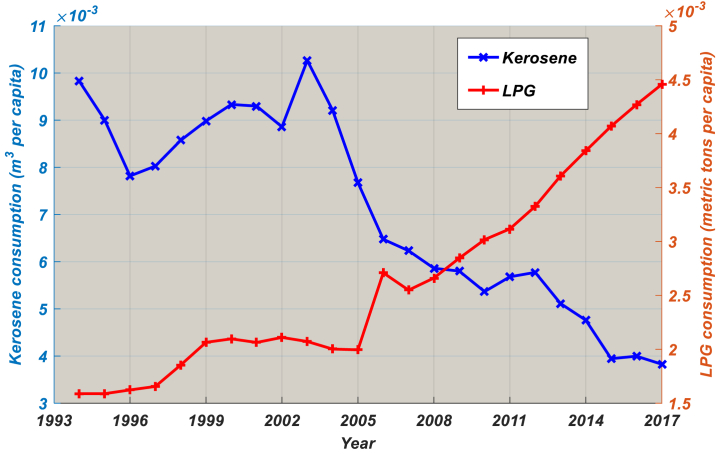
Historical LPG and kerosene consumption per capita, 1994–2017. Source: National Institute of Statistics [28]. Notes: Only LPG and kerosene supplied to households by marketers are taken into consideration.

Fig. 2 displays Cameroon's annual LPG consumption from 1994 to 2017, while Fig. 3 shows Cameroon's annual per-capita LPG consumption for the same period. Total LPG consumption and per-capita LPG consumption are both growing at high rates unlike Cameroon's average real GDP growth rate of 5–7%. With an average annual growth rate of 12.52%, domestic LPG consumption increased from 48,629 MT to 100,616 MT. With an average annual growth rate of 9.43%, LPG consumption per capita grew from 6.25 kg to 22.65 kg in 2017. Since the implementation of the National Energy Plan (NEP) in 1985, one of the objectives of which was to reduce kerosene consumption in favour of LPG (especially in rural areas), kerosene consumption has fallen sharply and LPG consumption took over in 2013 (Fig. 2). From 2009 to date, LPG consumption per capita has surpassed that of kerosene (Fig. 3), demonstrating the effectiveness of the NEP.

Cameroon's per capita LPG consumption currently stands at 28.43 kg per year, ranking first in Central African sub-region. In contrast to figures in Nigeria (46.38 kg per year), Angola (183.68 kg per year), Algeria (122.95 kg per year), and the European Union average (579.19 kg per year), Cameroon's per capita LPG consumption is still low [29]. Despite the low per capita consumption, the high overall consumption and fast growth rate in consumption point to a significant future increase in LPG use.

Table 1 displays LPG and kerosene demand by sector from 1995 to 2020. The highest portion of LPG consumption—74% of the total in 2020—is accounted for by the residential sector, which also includes households. This is justified by the rise in available income and the expansion of the number of households which grew by 50.29% between 2000 and 2005 and is anticipated to grow even more in the coming years. Only 2% of all LPG use in 2020 was accounted for by industries which reflects the low level of industrialization. Between 2001 and 2010, the industrial shares of kerosene consumption fell significantly, from 6.18% in 2001 to 4.6% in 2010. During the same period, the residential sector's share of LPG demand rose from 69.53% to 76.6% between accounting for the largest growth. In Cameroon, the use of the LPG in the transportation sector is extremely uncommon [22]. However, LPG is mainly used illegally in large trucks egines that dilute it with gasoline in order to benefit from the gross subsidy of LPG.

**Table 1:** LPG and kerosene consumption by sector.

Sector	1995		2000		2005		2010		2015		2020	
	LPG	Kerosene	LPG	Kerosene	LPG	Kerosene	LPG	Kerosene	LPG	Kerosene	LPG	Kerosene
Industry	1367,63	7750,40	2064,06	9186,38	2235,24	8599,72	2439,02	5028,90	5859,63	5679,73	6129,23	5689,56
Residential	15,386,99	87,198,27	23,222,32	103,354,26	25,148,31	96,753,78	47,592,04	78,630,65	65,925,56	63,901,55	97,460,46	64,012,10
Agriculture	663,90	3762,33	1001,97	4459,41	1085,07	4174,62	1863,60	3315,00	2844,48	2757,15	2946,23	2761,92
Transport	3419,09	19,376,00	5160,15	22,965,96	5588,11	21,499,29	6597,54	17,072,25	14,649,07	14,199,32	18,323,08	14,223,89
Commercial	442,60	2508,22	667,98	2972,94	723,38	2783,08	1242,40	2210,00	1896,32	1838,10	2630,82	1841,28
Power	849,79	4815,78	1282,52	5708,04	1388,89	5343,51	2385,41	4243,20	3640,93	3529,15	4051,17	3535,26
**Total consumption**^4^	22,130	125,411	33,399	148,647	36,169	139,154	62,120	110,500	94,816	91,905	131,541	92,064

Source: Ministry of Energy [27].

Notes: Animal husbandary, farming, fishing, forestry and water conservation are all part of the agricultural sector. Distribution, storage, and transportation are all included in the transportation sector. Wholesale trade and retail are both included in the commercial sector. Households and catering services are considered as part of the residential sector. LPG and kerosene consumption is in MT and cbm respectively.

**Fig. 4:**
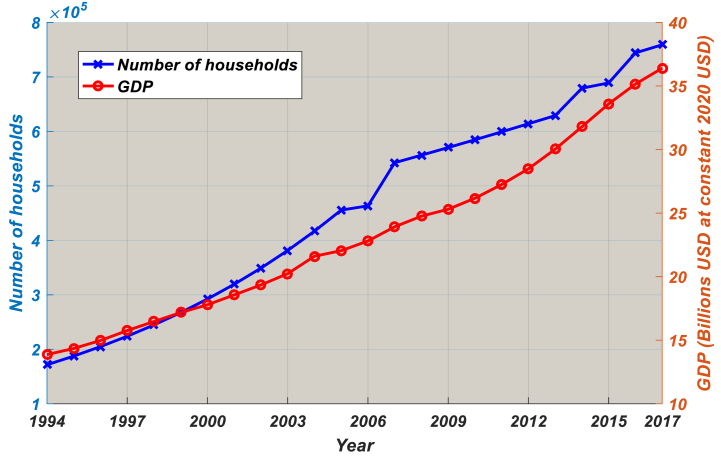
Total number of households having access to LPG and GDP, 1994–2017. Source: National Institute of Statistics [28].

One of the main causes of the rising LPG consumption in the household sector is the increase in disposable income, and this trend is anticipated to continue in the future [30]. The living standard in urban areas is growing very rapidly in Cameroon and has led to massive rural exodus over the two last decades. From 1985 to 2010, Cameroon's households grew rapidly, as shown in Fig. 4. Access to LPG increased from 463,331 homes in 2006 to 758,977 homes in 2017, with an average annual growth rate of 9.3%. The number of households in Cameroon is predicted to increase by 12–18% yearly between 2020 and 2030, reaching 2.5 million by 2035 [31]. Without an appropriate strategy, the share of LPG consumption in the household sector and overall LPG consumption will both be much higher than their current levels given the significant expected increase in the number of homes [32].

**Fig. 5:**
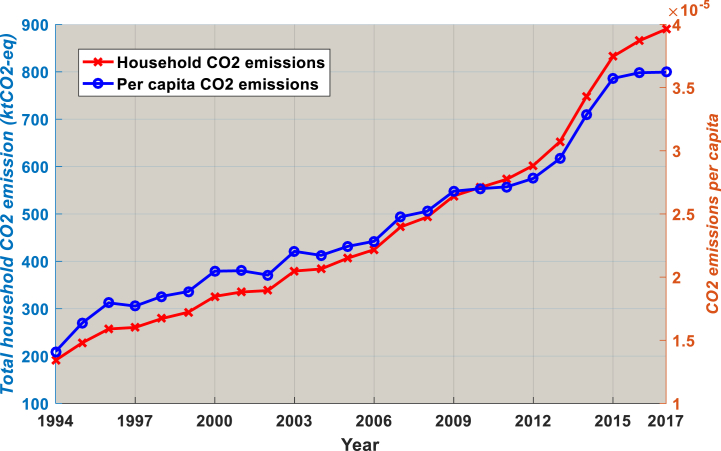
Total household CO2 emissions and CO2 emissions per capita, 1994–2017. Source: World Bank [36].

The increase use of OP in Cameroon has had negative repercussions, in addition to increased CO2 emissions, indoor pollution and excessive dependence on crude. Fig. 5 depicts the increase in overall CO2 emissions and per-capita CO2 emissions due to the use of LPG and kerosene between 1994 and 2017. Cameroon has overtaken all countries in the Central African sub-region to take first place since the early 2000s [33]. Engo [34] claim that in 2016, the residential sector accounted for 7% of all CO2 emissions; however, given the dramatic rise in number of households since then, the current percentage may be higher. Additionally, the risks to human health from indoor pollution have been increasing. High amounts of CO, VOC, and SO2 are found in homes, and hourly and daily levels of ozone and NOx have surpassed recommended air quality guidelines [1]. In some major cities, like Douala and Yaounde, indoor pollution ranks second as the primary cause of air pollution [35].

The present work examines the connections between the demand for LPG, disposable income, and its price. As contribution, this study is the first to calculate Cameroon's LPG demand elasticity using data gathered after 2000. The magnitude of the elasticity of demand for LPG is crucial for policy makers to assess the effectiveness of the 2017 fuel tax in regulating the use of OP. Our efforts to use comprehensive models and calculate an updated LPG elasticity at the country level, in our opinion, represent another essential contribution.

The remainder of this study is divided into the following sections: In Section 3, the econometric methodology is provided. Empirical results are exposed in Section 4. The discussion and implications for policy are presented in Section 5, while Section 6 delves into the conclusion with guidelines for future research.

## 3 Estimation of LPG demand elasticity

In this work, we use annual time series to estimate LPG demand elasticities in Cameroon's household sector. The methodology used in this study is the same as that used in the work of Lin and Zeng [37], Sapnken et al. [38] and Tamba et al. [39], except that we apply it to the household sector. The models used are: basic or static model, partial adjustment model (PAM), model incorporating interactions between drivers, model with macroeconomic variables, and error correction model.

The Douala University Institute of Technology ethical committee gave their approval and informed consent was obtained from all households. The methods employed in this investigation follow the guidelines laid out in the Declaration of Helsinki.

### 3.1 Basic or static model

We begin with a fundamental model as shown in Eq. (1) to calculate the income and price estimates of LPG consumption:

(1) $$D_{t}=\eta_{0}+\eta_{1}Y_{t}+\eta_{2}P_{t}+\eta_{3}U_{t}+u_{t}$$

Where $D_{t}$ is LPG demand, $Y_{t}$ is real per capita disposable income, $P_{t}$ is the real price of LPG, and $U_{t}$ represents the total share of urban population, which is used as a proxy for urbanization in this study. $u_{t}$ is a mean zero error term. The subscript t denotes the year. $Y_{t}$ and $P_{t}$ are both in constant 2020 USD. All variables in this study are in their natural logarithmic form. As a result, skewness and kurtosis are greatly reduced, and the coefficients can simply be translated as percentage variations [40,41].

The basic model's coefficients' interpretation is not entirely evident. The elasticities should be evaluated with Eq. (2):

(2) $$\frac{\partial D_{t}}{\partial Y_{t}}=\eta_{1};\;\frac{\partial D_{t}}{\partial P_{t}}=\eta_{2}\;and\;\frac{\partial D_{t}}{\partial U_{t}}=\eta_{3}$$

respectively. Hughes et al. [42] demonstrated that the majority of dynamic models typically provide longer-term elasticities that are larger than those of static models, which means that the estimates from a static model should be viewed as an intermediate-run elasticity (see Table 2). In order evaluate the sensitivity of the static model, we specify alternative models that handle the endogeneity of LPG pricing with regard to LPG consumption. Then, we consider the interaction effects between price and income, and incorporate other macro-economic variables into the model.

**Table 2:** Basic model.

Variable	Coefficient	Intermediate-run elasticities		
		Price	Income	Urbanization
Constant	0.692*	−0.897**	2.050**	8.953***
$Y_{t}$	2.050**	R-squared	86.67%	
$P_{t}$	−0.897**	Adjusted R-squared	85.25%	
$U_{t}$	8.953***	F-statistics	199	

*, **, *** respectively denotes significantivity at 5%, 1% and 0.1% thresholds.

### 3.2 Partial adjustment model (PAM)

We employ a PAM that allows demand at time t to depend on demand at time t−1 as well as income and price, in order to account for any market frictions that prevent adaptations to changes in LPG price and revenue from occurring in situ:

(3) $$D_{t}=\beta_{0}+\beta_{1}Y_{t}+\beta_{2}P_{t}+\beta_{3}U_{t}+\beta_{4}D_{t-1}+u_{t}$$

Eq. (3) is known as a partial adjustment model (PAM). The inclusion of lagged dependent variables increases the amount of data needed, but it also offers an easy method to take into account past variables that result in current changes in the dependent variable and are challenging to take into account in other methods. This model provides a lot of benefits especially when the series are integrated of order 1, I (1), as it then allows us to estimate both short- and long-run relations [43].

The presence of unit roots is checked for, and the variables' order of integration is determined, using the Phillips-Perron (PP) [44]. We employ PP test because it is more reliable than other tests in spotting unit roots [45]. Based on the PP statistics presented in Table 3, the existence of a unit root (null hypothesis) cannot be refuted at the 5% threshold for any of the series. The series are I (1) in nature, according to the results of the PP test performed on the first difference in the variables. Therefore, $\beta_{1}$ and $\beta_{2}$ stand for the demand for LPG's short-term income and price elasticities, respectively. Short-run estimates are then divided by $\left(1-\beta_{4}\right)$ to determine long-run elasticities as in Eq. (4):

(4) $$\gamma_{1}=\beta_{1}/\left(1-\beta_{4}\right);\;\gamma_{2}=\beta_{2}/\left(1-\beta_{4}\right)\;and\;\gamma_{3}=\beta_{3}/\left(1-\beta_{4}\right)$$

where $\gamma_{1}$, $\gamma_{2}$ and $\gamma_{3}$ are the respective long-run income, price and urbanization elasticities of LPG demand. Table 4 reports the results for the PAM model.

**Table 3:** Test of unit roots by Phillips-Perron (PP) for all the variables in this study.

Variables	Test equation	At levels	First differences
		PP test statistics	PP test statistics
$D_{t}$	Constant, trend	0.037	−5.923***
$P_{t}$	No constant, no trend	1.910	−3.475**
$Y_{t}$	No constant, no trend	1.563	−3.074**
$U_{t}$	Constant, trend	1.468	−4.985***
$G_{t}$	Constant, trend	1.082	−2.156*
$I_{t}$	No constant, no trend	1.510	−2.667*

*, **, *** respectively denotes significantivity at 5%, 1% and 0.1% thresholds.

**Table 4:** Summary of statistics, coefficients and estimation of elasticities for the PAM.

Variable	Coefficient	Elasticities					
		Short-run			Long-run		
Constant	−0.217	Price	Income	Urbanization	Price	Income	Urbanization
$Y_{t}$	0.107	−0.241	0.107	0.925	−0.315	0.823	7.115
$P_{t}$	−0.041	R-squared	98.11%				
$U_{t}$	0.925	Adjusted R-squared	97.63%				
$D_{t-1}$	0.870	F-statistics	292				

### 3.3 Model with interactions

Examining the relationship between income elasticity and price can be interesting at times. To achieve this, we build a regression model that incorporates a price-income interaction term as shown in Eq. (5):

(5) $$D_{t}=\alpha_{0}+\alpha_{1}Y_{t}+\alpha_{2}P_{t}+\alpha_{3}U_{t}+\alpha_{4}Y_{t}P_{t}+u_{t}$$

The partial effect of price on LPG demand holding income is:

(6) $$\frac{\partial D_{t}}{\partial P_{t}}=\alpha_{2}+\alpha_{4}Y_{t}$$

The coefficient $\alpha_{4}$ measures how receptive customers are to price changes in relation to how responsive they are to changes in income. If $\alpha_{4}>0$, the size of the price elasticity of LPG demand falls as income rises. We must test Eq. (6) at interesting values of $Y_{t}$, such as the mean value or the lower and upper quartiles in the sample, in order to summarize the impact of $P_{t}$ on LPG demand. It is frequently advantageous to reparameterize the model to provide an interesting interpretation for the coefficients on the original variables. We can reparameterize the model as:

(7) $$D_{t}=\theta_{0}+\theta_{1}Y_{t}+\theta_{2}P_{t}+\theta_{3}U_{t}+\pi_{4}\left(Y_{t}-{\bar{Y}}_{t}\right)\left(P_{t}-{\bar{P}}_{t}\right)+u_{t}$$

Where $\theta_{i}$ are the new coefficients, ${\bar{Y}}_{t}$ and ${\bar{P}}_{t}$ are the sample mean of $Y_{t}$ and $P_{t}$ respectively. We can identify the LPG price elasticity of demand at mean income by comparing Eqs. (5), (7); this yields Eq. (8):

(8) $$\theta_{2}=\alpha_{2}+\pi_{4}{\bar{Y}}_{t}$$

Table 5 presents the regression results.

**Table 5:** Demand model for LPG with price-income association.

Variable	Coefficient	Price elasticity
Constant	29.079	−0.330
$Y_{t}$	−3.218**	
$P_{t}$	−4.427*	
$U_{t}$	3.027*	
$Y_{t}\times P_{t}$	0.581***	
R-squared	81.63%	
Adjusted R-squared	79.92%	
F-statistics	307	

*, **, *** respectively denotes significantivity at 5%, 1% and 0.1% thresholds.

### 3.4 Model with additional macroeconomic factors

In addition to income and LPG prices, we would also like to consider other macroeconomic factors, such as the rate of urbanization $\left(U_{t}\right)\text{,}$ rate of inflation $\left(I_{t}\right)$, GDP $\left(G_{t}\right)$ and kerosene price ($K_{t})$ as shown in Eq. (9):

(9) $$D_{t}=\rho_{0}+\rho_{1}Y_{t}+\rho_{2}P_{t}+\rho_{3}U_{t}+\rho_{4}I_{t}+\rho_{5}G_{t}+K_{t}+u_{t}$$

By doing this, we examine the possibility that upward bias in elasticity estimations is caused by missing factors. Since the resumption of economic growth, factors including urbanization, inflation rates, and the cost of close substitutes like kerosene have been influencing variations in LPG consumption. If the economic crisis during the years 2001–2008 resulted in a fall in LPG demand, failing to take this effect into account would cause price estimates to be inflated. Table 6 displays the results of the regression in Eq. (9). The findings show that Cameroon's demand for LPG is significantly influenced by income, price, and urbanization.

**Table 6:** Model with other macroeconomic variables.

Variable	Coefficient	R-squared	67.13%
Constant	23.297	Adjusted R-squared	66.29%
$Y_{t}$	0.188**	F-statistics	258
$P_{t}$	−0.072*		
$U_{t}$	5.581***		
$I_{t}$	0.031*		
$G_{t}$	0.127*		
$K_{t}$	0.073*		

*, **, *** respectively denotes significantivity at 5%, 1% and 0.1% thresholds.

### 3.5 Error correction model (ECM)

The standard approach of an ECM makes the assumption that there is a functional connection between LPG consumption and important drivers like pricing, income, and urbanization [[46], [47], [48]]**.**

(10) $$D_{t}=\delta_{0}+\delta_{1}Y_{t}+\delta_{2}P_{t}+\delta_{3}U_{t}+u_{t}$$

Eq. (10) is at the basis of an ECM. Examining the stationarity of series constitutes an important step when defining an ECM. In general, a linear combination of the series $D_{t}$, $Y_{t}$, $P_{t}$ and $U_{t}$ is also I (1) if each series is separately I (1) [49,50]. The series might, however, possess a characteristic that causes a linear combination of them to become stationary. The series are referred to as cointegrated when this trait is present [49,50]. Long-term relationships exist between cointegrated series. The long-run elasticities of income, price, and urbanization are $\delta_{1}$, $\delta_{2}$ and $\delta_{3}$ respectively when the series are cointegrated. Finally, the ability to construct an ECM is enabled by the presence of a cointegrating function. To do this, the first differences between the variables are taken and all lags are incorporated as shown in Eq. (11):

(11) $${\Delta D}_{t}=\varphi_{0}+\sum_{i=1}^{L}\varphi_{1i}\Delta Y_{t-i}+\sum_{j=1}^{M}\varphi_{2j}\Delta P_{t-j}+\sum_{k=1}^{N}\varphi_{3k}\Delta D_{t-k}+\sum_{l=1}^{K}\varphi_{4l}\Delta U_{t-l}+\varphi_{5}{ECT}_{t-1}+u_{t}$$

Δ is the difference operator, whereas L, M, N and K are the lag lengths which are chosen using Hannan-Quinn, Schwarz, and Akaike information criteria, as described in Çetintaş [51]**.** $u_{t}$ is the serially uncorrelated error term, ${ECT}_{t-1}$ is the error correction term which captures the pace of compensation to its long-run equilibrium at the speed $\varphi_{5}$ whenever there is a drift. In other words, ${ECT}_{t-1}$ measures how quickly a divergence from the long-run equilibrium is corrected at time t−1. Finally, $\varphi_{11}$, $\varphi_{21}$ and $\varphi_{31}$ are the short-run elasticities of income, price and urbanization respectively.

## 4 Data and results

The annual time series data used in this analysis were collected from the HPSF, Cameroon's National Institute of Statistics, the IMF's International Financial Statistics, and were verified by the World Bank [36]. The data used in this work range broadly from 1994 to 2017. We chose annual data because there were no quarterly or other high-frequency data available. In addition, as an update for the sample period was not available, explanatory factors such as fuel taxes, oil shocks and the world market price of crude oil, could not be included.

According to economic theory, which states that demand decreases when product prices increase, price elasticities are likely to be negative. Income elasticities are expected to be positive since increases in income and demand are closely related. Finally, urbanization elasticities are expected to have a positive sign, meaning that as cities grow, less advanced traditional fuels such as kerosene and biomass will be replaced by more modern energies such as electricity and LPG.

### 4.1 Basic model

The basic model leads to consistent estimates and produces significant estimates of the intermediate-run price and income elasticity of −0.897 and 2.05 respectively, while intermediate elasticity of urbanization is 8.953. Although the results are in accordance with theory, price and income estimates seem to be very large (in absolute value). This explains why estimates produced by this model are considered as intermediate-run estimates [42,52].

### 4.2 PAM

The short- and long-run elasticities derived from the PAM are displayed in Table 4. We note that the estimates are all inelastic with urbanization having the largest impact on LPG demand. Consumers are highly inelastic in the short-run as price elasticity is not considerably different from zero. This makes sense given that changing consumption patterns, such as switching to alternative energies, which is often thought of as a long-run response, takes time, and customers have less time to adjust in the short-run. The model's findings demonstrate that there is a negative price effect on demand for LPG, thus indicating that it is a normal good.

Long-run elasticities are bigger in size than expected, although they are still less than unity. This demonstrates that with time, customers are more elastic. It should be mentioned that none of the calculated elasticities from the PAM model are significant due to the existence of the lag effects. Due to the LPG price's tendency to remain stable over time, these terms have a strong correlation with it. Since these correlations account for the majority of the variation in LPG consumption during the current period, the effects of price on LPG consumption are largely overridden. Additionally, the coefficient for the dependent variable that is lagged is extremely high (0.870), indicating a high level of persistence. In other words, the rate of adjustment is rather slow (around 13%).^5^ As a result of this low rate, there is inertia in the corrections from short-run deviations to the long-run equilibrium.

### 4.3 Model with interactions

The calculated value for price elasticity at mean income is −0.330. The price-income interaction term's significant positive estimate suggests that as income declines, customer sensitivity to LPG price increases rises. Consumers in Cameroon are responding more favorably to price increases due to the growing budget percentage of LPG consumption. This was demonstrated during the energy crisis of 2001–2008 when riots almost immediately broke out as the price of LPG was raised by 2.45 USD [25].

### 4.4 Model with other macroeconomic variables

As anticipated, the model incorporating other macroeconomic factors yields more inelastic pricing and income estimates of −0.120 and 0.146, respectively, when accounting for the impact of the energy crisis during the period 2001–2008. Both estimates are significant at the 5% level. The macroeconomic variables are individually insignificant but are jointly significant with F-statistic of 258. However, since the inflation rate is not very volatile, we give less weight to the macroeconomic model that incorporates it.

A positive (0.073), significant, but very close to zero cross price elasticity is seen in relation to kerosene. The explanation is that households' usage of kerosene as a substitute in response to LPG price increases is fairly limited. This shows that LPG is the preferred cooking fuel in most households (especially in the short-run) [25].

### 4.5 ECM

Johansen's cointegration test [53] results for the series $\left(D_{t},Y_{t},P_{t},U_{t}\right)$ are summarized in Table 7. One the one hand, Max-Eigen and Trace statistics permit the 5% level of significance rejection of the null hypothesis that no cointegrating equation exists (R=0). On the other hand, at the 5% level of significance, we accept the null hypothesis (R≤1) of existence of a maximum of one cointegrating equation. This suggests that there is just one cointegrating equation, $D_{t}=0.352+0.569Y_{t}-0.401P_{t}+18.624U_{t}$, which is found to be significant at the 5% level. Thus, the long-run elasticities of income, price and urbanization are 0.569, −0.401, 18.624 respectively. We note that LPG demand is inelastic for both income and price in the long-run, whereas urbanization has the highest impact on demand for LPG in Cameroon.

**Table 7:** Results for the ECM.

Johansen cointegration tests				
Null Hypothesis	Max-Eigen Statistics	P-values	Trace Statistics	P-values
None a (R=0)	29.165	0.75%	59.871	0.26%
At most 1 (R≤1)	18.350	13.09%	30.104	9.19%
	Elasticities			
	Short-run	Long-run		
Income	0.159**	0.569***		
Price	−0.117*	−0.401**		
Urbanization	5.025***	18.624***		

Note: Using AIC, SIC, and HIQ, the ideal lag length is found to be two.

R is the number of cointegrating equations.

^a^Denotes rejection of the null hypothesis at the 5% level.

*, **, *** respectively denotes significantivity at 5%, 1% and 0.1% thresholds.

According to estimates, the demand for LPG has a short-run price and income elasticities of −0.117 and 0.159 respectively (Table 6). Both have a 10% and 5% threshold of statistical significance, respectively. The signs are in accordance with theoretical expectations, and demand for LPG is both income and price inelastic in the short-run. Once more, we note that urbanization influences LPG consumption the most in the short-run. Furthermore, the ECT, which gauges how quickly short-run deviations are corrected to long-run equilibrium, is −0.232. Thus, with only 23.2% of the entire adjustment taking place within the first year, this indicates that LPG demand adapts towards its long-run equilibrium.

**Fig. 6:**
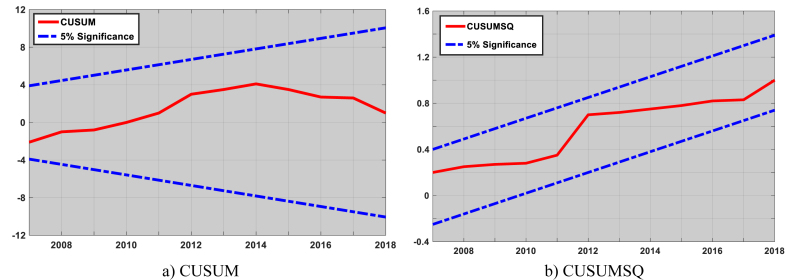
Cumulative sum of recursive residuals.

Testing the model's accuracy is crucial when utilizing cointegration approaches because any misspecification could create instability. In this study, we employ CUSUM^6^ and CUSUMSQ^7^ to test for model specification and to account for variables that might be excluded but are still important to the model [46,49]. Fig. 6 (a,b) demonstrates that the 95% confidence interval is not exceeded by the CUSUM and CUSUMSQ plots. Consequently, we conclude there is no structural break as stipulated by the null hypothesis. In light of this, the model is stable and, more significantly, no significant explanatory variable has been left out.

## 5 Discussion of results and policy implications

The price elasticity estimates produced by the price-income interaction and ECM models are significant and fall between −0.330 and −0.401. The estimates of the income elasticity of LPG demand produced by these two models are also reliable and substantial, and they fall between 0.159 and 0.569. The inclusion of lag variables tends to drown out the impacts of income and price on LPG demand in the current period, hence we are less confident in the PAM model.

Price elasticities are statistically different from zero and less than unity in the short-run, showing that households are quite inelastic in the short term. This shows that households either cannot opt for an alternative source or that they have a greater preference for LPG. Income elasticities indicate that LPG demand is also income inelastic in the short-run. This proves that LPG is a typical good and that, in the short-run, an increase in income does not significantly boost LPG demand.

Results show that Cameroon's long-run demand for LPG is significantly influenced by income, price, and urbanization. Elasticities have larger magnitudes in the long-run than their corresponding short-run estimations. Nonetheless, the estimates are still less than unity. This may show the lack of available substitutes. The higher long-run income elasticity compared to the short-run estimates indicates that households are consuming at the limit they would need or that they would prioritize other consumption goods than energy (this is particularly true for very poor households). Our findings are confirmed by the works of Akpalu et al. [54]**,** Mensah [47] and Mensah et al. [48]**.**

Finally, the estimates of urbanization's elasticity are both significant and very elastic, in contrast to those of income and price. This is not surprising because most of the LPG retail outlets are found in metropolitan regions, which represents more than 80% of Cameroon's LPG users by 2020 [1]**.** The choice for more contemporary and effective energy sources like LPG is also influenced by the rising standard of living in urban areas. Therefore, the emerging demographic transition to urban settlement exerts significant influences on LPG demand in Cameroon. These outcomes match the works of Mensah [47] and Mensah et al. [48] for Ghana**,** and have significant policy implications with regard to LPG demand in Cameroon.

Due to recurrent supply shortages and the fact that demand for LPG exceeds production capacity, it is essential to make large expenditures in order to speed up the growth and modernisation of SONARA, the country's unique refinery. As a result, this refinery will be able to boost its refining capacity from 2.1 to 3.5 million MT by 2025. Also, LPG storage capacities (12,584 cbm in 2020) must be increased, notably in regions where there are no reserves. This will safeguard the nation's strategic supplies, avert any impending shortages, and maintain adequate supply on the domestic market. Liberalizing the oil industry will make marketers more influential in the structure of the LPG market, which will be effective in addressing irregular shortages. Competition would undoubtedly result from this.

Due to seasonal roadways and the hilly topography, rural communities are underserved. Modernizing transportation logistics and enhancing present modes of transportation are both necessary. There may be other options, such as rail transportation [55]. By constructing pipeline infrastructure to transfer OPs from production facilities to end consumers, transportation costs can be reduced over the long-run, contributing to a sustainable supply of LPG. This has shown to be the most cost-effective mode of fuel transportation, despite the substantial investments required [56].

LPG should be subsidized in its conditioned form rather than in its bulk form to prevent industries and carriers from profiting from the subsidy. To ensure that carriers are not eligible for the subsidy, precautions must be taken. For instance, redirecting the LPG subsidy to its end-use equipments, such as tubes, relief valves, stoves, cylinders, etc., will be a different way to solve the “unintended beneficiary” conundrum. These measures, for example, were effective in Ghana in removing a significant number of *unintentional beneficiaries* [48].

Given that LPG consumers are sensitive to both its price and substitute, pricing mechanism can be used to encourage a switch from less ecologically friendly energy sources like kerosene to cleaner and more efficient sources like LPG. The findings from this study reveal that estimates of price elasticity are less than unity, which denotes that families' reactions to price fluctuations are comparatively insignificant. However, given the high degree of substitutability and the overall positive impacts of higher energy costs, increases in the price of oil products must be done gradually to prevent any potential spillover effects.

It is impossible to continue the current economic system, which is built on limited and scarce resources [57]. OP are nonrenewable resources that will eventually run out. Additionally, they produce GHG, which hastens the global phenomenon of climate change. Therefore, it is appropriate for the government of Cameroon to start investigating several possible alternative and greener energy options. Moving to alternative energy sources would need a significant initial financial outlay, a lot of labor, and time. The State of Cameroon must plan its energy use while taking alternative energy sources into consideration if it wants to maintain energy security and continue the country's economic growth in the next years.

## 6 Conclusion and direction for future research

In this study, LPG elasticities for Cameroon were estimated using annual time series data from 1994 to 2017. For this, a basic model and four alternative specifications models were used. An ECM model was equally determined after testing for stationarity and the presence of a cointegrating function. The results obtained were as follows: the series are all I (1); there exist a cointegration relationship; the established ECM is the most robust of all specified models as it produces significant estimates of income, price and urbanization elasticities. The reliability of the model specification was verified using the CUSUM and CUSUMSQ.

Price, income, and urbanization are key factors of LPG consumption in Cameroon, according to the results of the models mentioned. In addition, the models show that while urbanization and income have a positive impact on LPG consumption, market prices have a negative impact on demand. More so, urbanization is estimated to have the strongest elasticity on the long- and short-run dynamics of LPG consumption. This indicates that the growing urban population is mostly to blame for the continued rise in LPG usage in Cameroon, which frequently results in supply shortages. These outcomes are consistent with the works of Mensah [47] and Mensah et al. [48].

Another interesting finding is the high level of inter-fuel switching in OP use in Cameroonian households. The models' cross-price elasticities are significant, which is proof of this. This study's data support the idea that switching from kerosene to LPG usage can be done to a large extent. This is mostly a result of Cameroon's LPG subsidies, which have led to a rise in LPG use by industries. In other words, despite environmental motivations to reduce deforestation and CO2 emissions, LPG use is expanding in Cameroon mostly due to its efficiency and economic advantages over conventional fuels. The existing Cameroon subsidy policy has to be carefully reviewed in light of this.

The government of Cameroon therefore needs to take both short- and long-run measures to sustain the expansion of LPG use. Modernisation and growth of SONARA should not be delayed. The use of fuelwood, which contributes to desertification and deforestation must be limited in favour of LPG. The HPSF should continue to support SONARA's LPG production. There is a need to liberalise LPG distribution, marketing and transport in order to promote widespread LPG use. Cameroon's gas fields should also be used to improve LPG supply, given the country's scarce crude oil reserves and growing household demand for LPG.

In order to improve this work, future research could try using higher frequency data and, above all, ensuring that there is sufficient variation in the series, as there is a risk that unobserved factors, such as intrinsic changes in household fuel preferences, could distort the results. Also, although we have studied income and price elasticities of LPG demand, we have not considered the potential impact of subsidy and tax adjustments. Future research could address this by simulating a simple policy of the effect of subsidy or tax modifications. Furthermore, a detailed analysis should be conducted to fully assess the costs and benefits of changes in Cameroonian household energy use. Despite these limitations, the present study contributes to a better understanding of household responses to LPG price and income changes and the possible impact of subsidy and tax policy options.

## Author contribution statement

Flavian Emmanuel Sapnken: Conceived and designed the experiments; Performed the experiments; Analyzed and interpreted the data; Contributed reagents, materials, analysis tools or data; Wrote the paper.

Marius Tony Kibong: Conceived and designed the experiments; Analyzed and interpreted the data.

Jean Gaston Tamba: Conceived and designed the experiments; Analyzed and interpreted the data; Wrote the paper.

## Funding statement

This research did not receive any specific grant from funding agencies in the public, commercial, or not-for-profit sectors.

## Data availability statement

Data will be made available on request.

## Additional information

No additional information is available for this paper.

## Declaration of competing interest

The authors declare that they have no known competing financial interests or personal relationships that could have appeared to influence the work reported in this paper.

## Footnotes

1 Only kerosene, considered the preserve of the poorest populations, is not affected by this reform.

2 Some of the marketers include TOTAL, ALGO, ALPHA OIL, BOCOM, OLMEX, MRS, OLA, TRADEX, GULFIN, GREENOIL, DELTA PETROLEUM and NEPTUNE.

3 1650 MT in spheres and 3817 MT in butane are available in Douala and in Yaoundé, there is a stock of 108 MT. In addition, the Bipaga centre in the south of the country continues to deliver products for marketers who do not load at SCDP.

5 Only 13% of the difference between equilibrium LPG use and actual consumption is reduced annually.

6 CUSUM stands for CUmulative SUM of recursive residuals.

7 CUSUMSQ stands for CUmulative SUM of SQuared residuals.

## Footnotes

4 It should be noted that the totals shown here differ from the total consumption for all 10 regions in Table 1. The data in Fig. 2 and Table 1 are from the Cameroon Ministry of Energy. Although Fig. 2 shows total LPG and kerosene consumption for the ensemble of 10 regions, Table 1 includes LPG and paraffin consumption by sector. The Ministry of Energy compiles both sets of statistics from a variety of sources, which explains much of the disparity. As it is not possible to fully cover all sectors, LPG and kerosene consumption by sector is certainly underestimated. However, we have included Table 1 to show the respective shares of each industry as well as their variations.
